# Evaluation of Random and Aligned Polycaprolactone Nanofibrous Electrospun Scaffold for Human Periodontal Ligament Engineering in Biohybrid Titanium Implants

**DOI:** 10.1155/2024/2571976

**Published:** 2024-10-17

**Authors:** Ihab N. Safi, Basima Mohammed Ali Hussein, Aseel Mohammed Al-Khafaji, Abdalbseet A. Fatalla, Ahmed M. Al-Shammari

**Affiliations:** ^1^Department of Prosthodontics, College of Dentistry, University of Baghdad, Baghdad, Iraq; ^2^Department of Biomedical Applications, Institute of Laser for Postgraduate Studies, University of Baghdad, Baghdad, Iraq; ^3^Experimental Therapy Department, Iraqi Center for Cancer and Medical Genetic Research, Mustansiriyah University, Baghdad, Iraq

**Keywords:** dental implant, electrospinning technique, periodontal ligament, poly(caprolactone) nanofibrous, stem cells

## Abstract

**Background:** Stem cells are introduced to regenerate some living tissue to restore function and longevity. The study aims to isolate in vitro human periodontal ligament stem cells (hPDLSCs) and investigate their proliferation rate on plasma-treated aligned and random polycaprolactone (PCL) nanofibrous scaffolds made via an electrospinning technique to attempt periodontal-like tissue in dental implants.

**Materials and Methods:** hPDLSCs were isolated from extracted human premolars and cultured on plasma-treated or untreated PCL-aligned and random scaffolds to enhance adhesion of periodontal ligament (PDL) cells as well as interaction and proliferation. Cell morphology, adhesion, and proliferation rate were evaluated using field emission scanning electron microscopy (FESEM) and the methyl tetrazolium (MTT) assay. The wettability of PCL scaffolds was tested using a goniometer.

**Results:** The hydrophilicity of plasma-treated scaffolds was significantly increased (*p* ≤ 0.05) in both aligned and random nanofibers compared to the nontreated nanofibrous scaffold. Cells arranged in different directions on the random nanofiber scaffold, while for aligned scaffold nanofibers, the cells were arranged in a pattern that followed the direction of the aligned electrospun nanofibres. The rate of hPDLSC proliferation on an aligned PCL nanofiber scaffold was significantly higher than on a random PCL nanofibrous scaffold with a continuous, well-arranged monolayer of cells, as shown in FESEM.

**Conclusion:** The aligned PCL nanofiber scaffold is superior to random PCL when used as an artificial scaffold for hPDLSC regeneration in PDL tissue engineering applications.

## 1. Introduction

In the presence of damaged and periodontally injured teeth, repairing the periodontal ligament (PDL) may be a primary issue for periodontal tissue regeneration. Numerous methods were used for periodontal tissue regeneration, including artificial scaffolds, bioactive materials, and tissue replacements [1]. Because nano-biomaterials are mostly used as scaffolding and delivery systems, there is a greater demand for research on biomaterials in living organisms. The advantage of nano-biomaterials has spurred more research into fields like scaffold production [2]. With the variety of production sources that may serve biocompatibility and improve living tissue tolerance [3], tissue engineering scaffolds must satisfy many requirements, like being biocompatible, biodegradable, et cetera. Motivated by the design of native extracellular matrix (ECM), fibrous scaffolds were viewed as a committed approach for designed tissue substitutes because they offer advantages, including the fibers themselves allowing for a wide expanse for the adhesion of the cells, high interfibre spacing that allows for cell infiltration, nutrition, and gas exchange, and adaptable structural support for the requirements of the tissue being replaced. Increase the surface-to-volume proportion, which will enhance interactions between cells and scaffolds [4, 5]. The scaffold seeded with cells in tissue engineering performs better because the size and three-dimensionality of those fibers mimic the ECM, which improves the adhesion of cells to scaffolds, encourages cell migration, and promotes cell growth [4–9]. Nanofibers are one of the most intensively researched nanomaterials for regeneration tissue, created using various processes such as electrospinning, self-assembly, phase separation, and drawing [10]. Hyaluronic acid, alginate, fibrin, collagen, and gelatin are examples of natural nanofiber scaffolds [11]. Polycaprolactone (PCL), polyethylene glycol, and polylactic acid (PLA) are the most common synthetic scaffolds [12]. PCL is a polymer with a storied record of risk-free use in people and food and drug administration (FDA) approval [8]. In biomedical engineering and regenerative medicine, all systems used in drug release or implantation of materials or biomaterials should have primary features to be included or used safely and without any possibility of adverse effects or failure factors related to the material. These are unique in their mechanical properties to match the structure used; they also have to have miscibility, potency, and biodegradation properties [13]. This biomaterial is both biocompatible and biodegradable and exhibits a wide spectrum of hydrophobic properties [9, 14, 15]. For these reasons, this material was used in different applications, including areas of highly precise function and specialized structure, such as in heart failure conditions, to replace myocardium [16]. The flexible properties of this material made it able to be used in different forms as it was combined with polymers and hydrogels to enhance its properties or to develop new PCL-based composites for bone tissue engineering [17]. These modifications may overcome the poor hydrophilia and low bioactivity of pure PCL systems and expand the limits of their applications in the biomedical field [18].

In the past 10 years, electrospinning has become a straightforward and effective method for producing nanoscale fibers with a diameter ranging from a few nanometers to very few microns [4]. Until now, electrospinning has just been accomplished with more than 100 different polymers, including regularly used biodegradable synthetic polymers like PLA [5], poly(glycolic acid) (PGA) [6], poly (lactide-co-glycolide) (PLGA), and PCL [7]. Electrospun natural polymer fibers, including silk, chitosan, and collagen, have also proven successful [8].

Sources of stem cells such as those derived from the periodontal ligament (PDLSCs) are extremely valuable and trusted for the renovation of the tissue of the PDL in both animal and human trials; therefore, the PDL can surely be utilized as a provenance of stem cells [19]. Furthermore, the collection of stem cell properties and PCL with biomaterials using different techniques introduced a new era in dental implant technology, and the introduction of biohybrid design in an attempt to overcome shortages appeared with the use of conventional implant materials and procedures [20].

The idea that different alignments of the nanofibers may affect stem cell adhesion and growth rate is an alternative to the hypothesis that nanofiber silhouette may not affect the success of cell adhesion and growth in this study. In this study, plasma method was utilized to treat the scaffolds' surfaces, making them more receptive to human periodontal ligament stem cells (hPDLSCs).

## 2. Material and Methods

### 2.1. Isolation and Culturing of hPDLSCs

The Human Ethics Committee at the College of Dentistry, University of Baghdad (ID: 822223) gave approval for this study. All of the donors provided their written consent. Premolars (35) were extracted during orthodontic treatment for patients' age range (13–26 years) at the Oral and Maxillofacial Unit of the Academic Dental Hospital/College of Dentistry, University of Baghdad. The teeth were used in this study during the period of time between October 1^st^ and December 1st, 2021. The authors have selected the most significant primary articles through PubMed and Google Scholar searches and provided an overview of the topic's most significant developments.

The study excluded participants with periodontitis, gingivitis, caries, and periapical lesions. A local anesthesia was used during the dental extraction. Immediately after extraction, the extracted teeth were put in free media at 4°C and in transport phosphate-buffered saline (PBS) media (Chemical Point, Germany), that contained 100 units/ml of penicillin, 100 units/ml of streptomycin, and 100 units/ml of amphotericin (Capricorn-Scientific, Germany), petri dishes were used to contain these teeth temporarily in cell culture laboratory under a sterilized and laminar flow hoods with heap filters (media Capricorn, USA) till steps of processing and culturing was performed. Premolar teeth were cleaned five times; the entire process was 5 min with PBS that contained antibiotics. Scraping of the roots was done using surgical scalpel in the preparations for PDL collection. It was planned to have a single cell suspension; therefore, all obtained PDL tissue in the test tube that containing pure minimum essential medium (MEM) was blended with 0.75 g/ml collagenase/dispase (Sigma–Aldrich, Germany) at 37°C with rapid shaking for 7 h. The test tube that contained the cell suspension was spin at 1500 rpm for 3 min at 17°C in a centrifuge (Hettich, Germany). After removing the top media layer, the test tube was filled with 8–10 ml of the MEM contains 20% fetal bovine serum (FBS) culture. The suspension was cultivated and stored at 37°C in a Memmert incubator with 5% CO_2_. After 7 days, the medium was changed to MEM with freshly added 12.5 ng/ml fibroblast growth factor (FGF2, US-Biological, USA), 20% FBS (Capricorn-Scientific, Germany) and 80 g/ml ascorbic acid (US-Biological, USA) [21]. Every 3 days, the medium was changed. The cells had approximately 80% proliferation on day 21.

### 2.2. Immunofluorescence Assay

Chambers of a tissue culture filled by MEM with 20% FBS were seeded with 5 × 10^5^ of hPDLSCs [22]. Aspiration of the medium was done, and the cells were fixed in cold acetone (Chemical Point, Germany) for 3 min before being left to dry. After 5 min of PBS rinse, the slides were incubated with a 4% blocking reagent (Santa Cruz Biotechnology in the United States) for 1 h to block nonspecific binding before receiving a final 5-min PBS quick rinse. The cells were then washed with PBS for 5 min before being immunostained with a 1:50 periostin antibody (Santa Cruz Biotechnology, USA) overnight at 4°C. An inverted fluorescence microscope was used to examine the slides in a dark field after the final step of cover slide fixation was done using glycerin 50% and PBS 50%.

### 2.3. Hematoxylin and Eosin (H&E) Staining

H&E stains were used to study the morphology, including nuclear as well as cytoplasmic.

• PDLSCs were cultured on a cover slide in a six-well tissue culture plate at 5 × 10^5^ for each well; aspiration of the medium was done after 3 days. Fixation of the cells was done by adding 10% formaldehyde (diluted in PBS) for 30 min, then washed two times using distilled water (D.W.). Dehaydration of the cells was done by using ethanol alcohol (100%, 90%, and 70%). Cells were kept in each concentration for only 2 min and finally washed two times using distilled water. To study cell content and structure, hematoxylin stain was used for 20 min to identify the nuclear contents, then washed again with distilled water two times. Eosin stain was used to identify cytoplasmic content for 2 min and was also washed with distilled water two times. Dehydrated in absolute ethanol at 100% for 5 min. Finally, the cells were mounted with dibutylphathalate polystyrene xylene (DPX) and photographed with the camera of the optical microscope.

### 2.4. Characterization of Nanofibers

#### 2.4.1. Study of the Morphology

The prepared PCL nanofibers, random or aligned, were examined by inverted microscopy (scanning electron microscope [SEM]; XL30; Philips, Eindhoven, Netherlands).

#### 2.4.2. Surface Modification of Nanofibers

Plasma surface modification of PCL scaffold discs was performed using a Diener electronic plasma cleaner (Germany) to increase the hydrophilicity of the scaffold. Low-pressure oxygen-plasma pretreatment was done, and the plasma discharge was applied at 30 W under vacuum mode in the plasma chamber for one cycle/3 min. Nitrogen and NH_3_/helium were then introduced into the device at a flow rate of 150 cm^3^/min.

#### 2.4.3. Contact Angle Measurement

After surface modification, the hydrophilicity of the nanofibrous was evaluated at room temperature using a contact angle goniometer (Krüss, Hamburg, Germany). Its principle of use is to measure the contact angle of a distilled water droplet with a surface. At a fixed distance and droplet volume, the contact angle was measured after 10 s; the contact angle between a water droplet and the nanofibers was evaluated.

### 2.5. Scaffold Seeding and hPDLSC Proliferation

A flask was used to establish cell cultures, which were then incubated at 37°C in a humidified incubator with 5% CO_2_. Every 3 days, the medium was replaced, and a fresh FGF2 12.5 ng was added with each media change. Trypsinization was used to collect the cells once they were between 80% and 90% confluent [23].

PCL scaffold discs, 12 mm in diameter (Z694533 Nanofiber multiwell plate, with aligned nanofibers for 24-wells, and Z694525 Nanofiber multiwell plate inserts, with randomly oriented nanofibers for 24-well plates, Sigma–Aldrich), were placed in 24-well flat-bottom labeled plates containing 500 µl of MEM with 20% FBS and cells of the PDL were seeded at a density of 100,000 cells per well.

### 2.6. Evaluation of a Random and Aligned Polycaprolactonen Nanofibrous Electrospun Scaffold for Human PDL Engineering

Culturing of the cells on both prepared types of PCL nanofibers (random and aligned) was done in 24-well cell culture plates and studied by inverted microscopy (SEM; XL30; Philips, Eindhoven, Netherlands), methyl tetrazolium (MTT) assay, and field emission scanning electron microscopy (FESEM) to evaluate the tested scaffolds.

#### 2.6.1. MTT Assay for hPDLSC Proliferation

According to ISO 10993-Part 5, the proliferation rate of PDLSCs was evaluated in vitro using the MTT test for cytotoxicity and biocompatibility on both randomly placed and oriented nanofibrous scaffolds. hPDLSCs were seeded at a cell density of 10,000 cells per well for the MTT assay. The culture medium was removed from the wells of the plates after the incubation times, and the viability of the cell cultures was determined using a MTT (Santa Cruz, USA) solution cytotoxicity test. One hundred microliters of a fresh MTT solution containing 5 mg/ml was added to each well in a dimly lit area. The plates were then left to incubate for 4 h. MTT was transformed into formazan crystals by the mitochondrial dehydrogenase of active PDLSCs. The supernatant was removed, and each well received 50 µl of dimethyl sulfonic oxide (DMSO; Santa Cruz, USA). After transferring the DMSO solution to new 96-well flat-bottom plates, the optical density at 570 nm was measured using an automated microplate-based multidetection reader (FLOUstar OPTIMA, microplate BMG LABTECH, Germany). A triplicate model was used for every experiment.

### 2.7. Statistical Analysis

Data were analyzed using Statistical Package for Social Science, version 21 (SPSS), and Prism 9 (GraphPad Software, USA). Results were presented in a bar chart to display the means and standard deviations (descriptive analysis). One-way analysis of variance (ANOVA) and the *t*-test were used for the multiple comparisons. Nonsignificant (NS), significant (S), and highly significant (HS) differences were defined, respectively, as *P* values larger than 0.05, less than 0.05, and less than 0.01, respectively. The authors have selected the most significant primary articles through PubMed and Google Scholar searches and provided an overview of the topic's most significant developments.

## 3. Results

**Figure 1:**
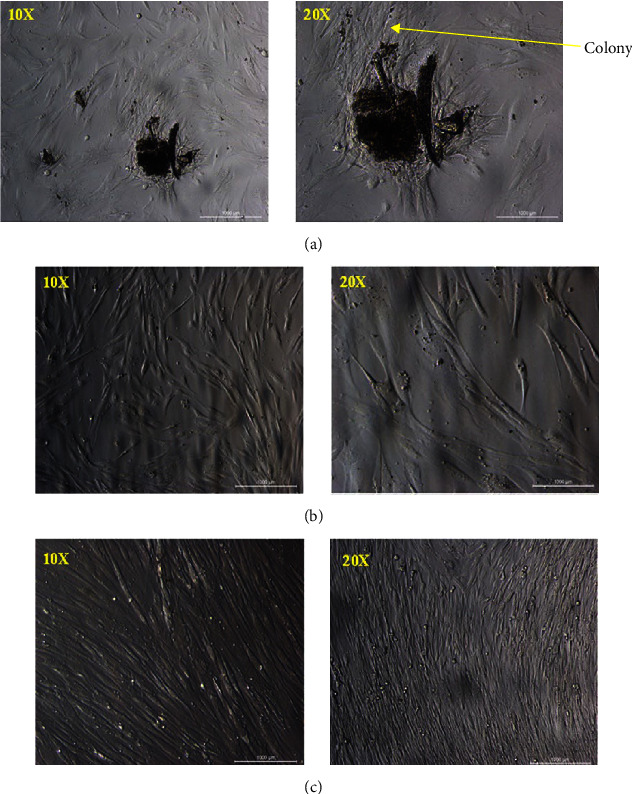
Inverted microscope images for hPDLSCs (scale bars: 1000 µm): (A) 6 days, (B) 15 days, and (C) 22 days. hPDLSCs, human periodontal ligament stem cells.

Under the microscope, the cells of the primary culture appeared as either single or cluster cells, as shown in (Figure 1A–C). The PDLSCs, shaped like spindle cells, were identified in small colonies that were well adherent and appeared after 7 days of cultivation. The medium was changed to get rid of nonadherent cells. The cells start to proliferate and form a confluent monolayer on days 8–20, forming spindle-shaped fibroblasts. Colonies with diameters ranging from 0.5 to 3 mm reached 80% confluency in 21 days.

**Figure 2:**
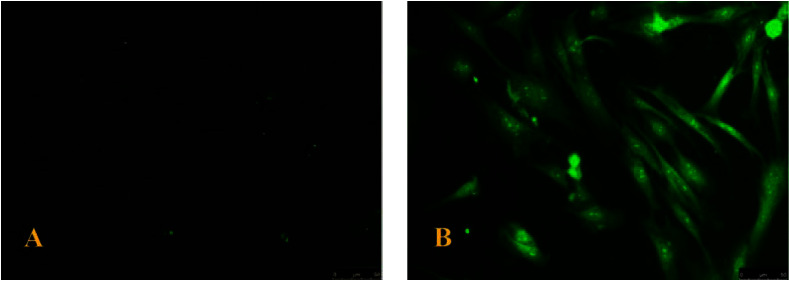
Periostin interaction with hPDLSCs under fluorescence microscopy at 20x (scale bars: 500 µm). (A) Negative expression. (B) Positive green expression. hPDLSCs, human periodontal ligament stem cells.

Immunofluorescence phenotyping of the cultured PDLSCs showed that more than 90% of the PDLSCs were stained positive for periostin (positive green expression) compared to control hPDLSCs that did not show any expression (negative expression), as shown in (Figure 2).

### 3.1. H&E Staining

**Figure 3:**
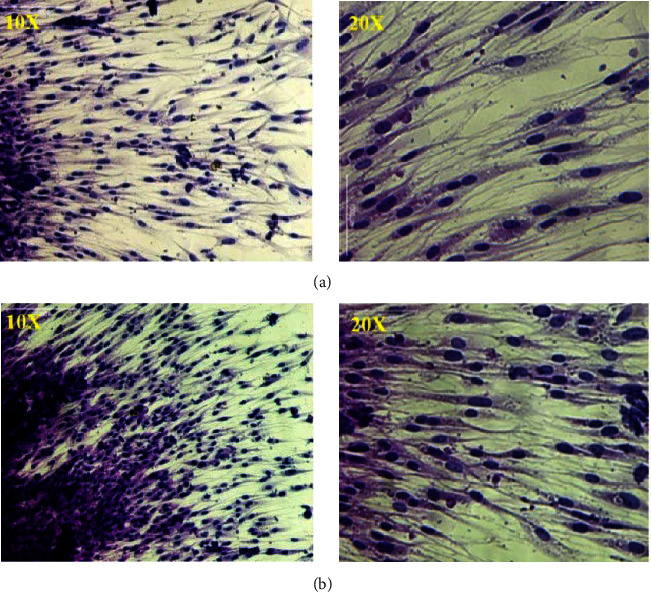
Inverted microscope images for hPDLSCs (P0) at 10x and 20x (scale bars 1000 µm): (A) day 15 and (B) day 22. hPDLSCs, human periodontal ligament stem cells.

Morphological results by H&E staining showed the PDLSCs are spindle, long, and thin (60–80 μ), as shown in (Figure 3).

### 3.2. Characterization of Nanofibers

#### 3.2.1. Study of the Morphology

**Figure 4:**
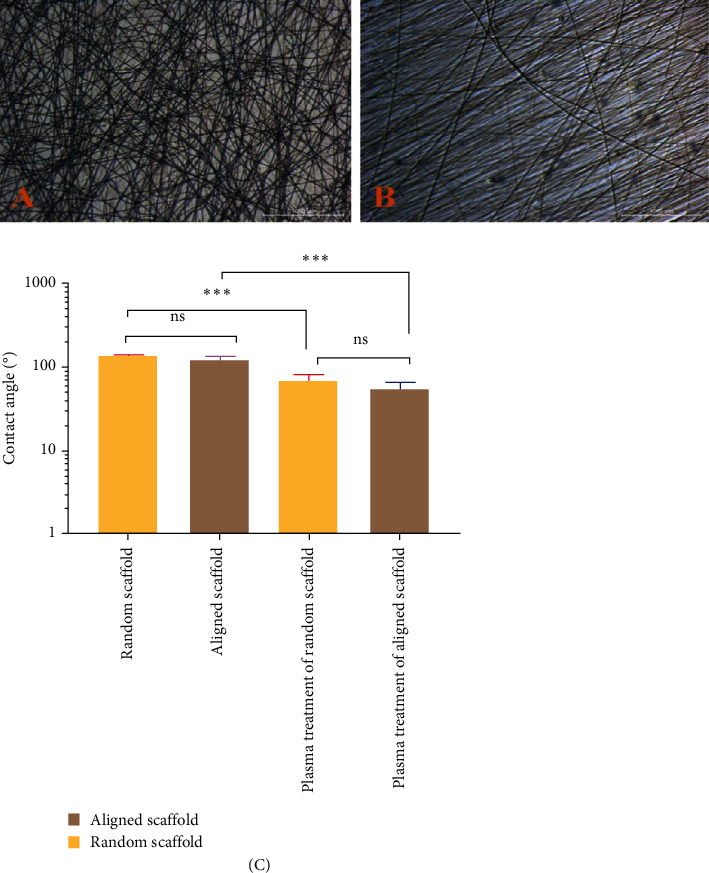
Inverted microscope images of (A) a random scaffold, (B) an aligned scaffold (scale bars 1000 µm), and (C) contact angle (°) for random and aligned scaffolds before and after plasma treatment. *⁣*^*∗∗∗*^*P* = 0.0001. ns, not significant.

Figure 4A,B shows the PCL scaffolds that were prepared by the electrospinning technique, illustrating either the aligned or random orientation of the fibers.

The results obtained from measuring the contact angle indicated that the wettability (hydrophilicity) of plasma-treated scaffolds was significantly (*p* ≤ 0.05) higher in both aligned and random nanofibers in comparison to nontreated nanofibrous scaffolds, whether aligned or random (Figure 4C).

### 3.3. Cell Proliferation

**Figure 5:**
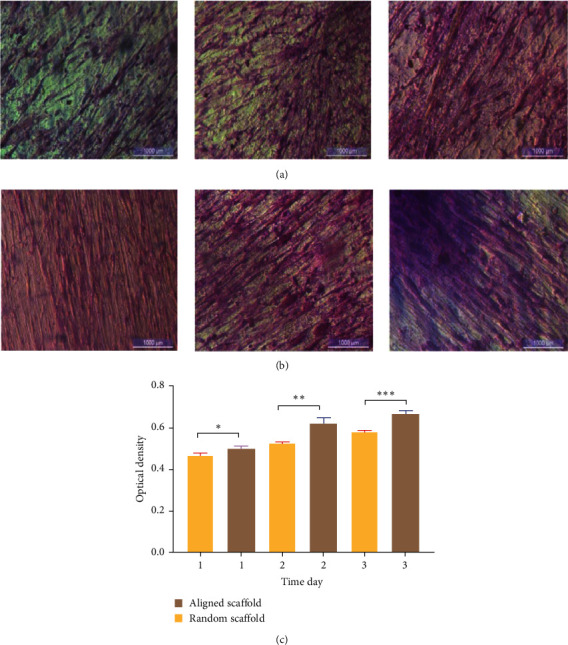
Inverted microscope images (scale bars 1000 µm): (A) of seeded random PCL scaffold with higher cell proliferation in the time intervals, (B) of seeded aligned PCL scaffold with progressed proliferation of the cells in time intervals, and (C) proliferation rate on random and aligned scaffolds during a 3-day cell culture period. PCL, polycaprolactone. *⁣*^*∗*^*P* = 0.0143. *⁣*^*∗∗*^*P* = 0.0017. *⁣*^*∗∗∗*^*P* = 0.0002.

The proliferation of PDL on aligned PCL nanofibrous electrospun was higher than the cell proliferation on random PCL nanofibrous electrospun for human PDL engineering (Figure 5A,B).

The MTT assay results showed that the proliferation of cells was significantly (*p* ≤ 0.05) increased in aligned nanofibers compared to random nanofibrous scaffolds (Figure 5C).

**Figure 6:**
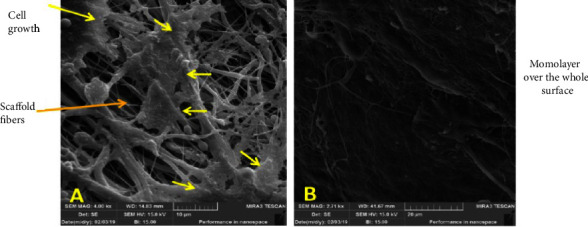
FESEM image of seeded scaffolds at 22 days. (A) Seeded random PCL scaffolds with low growth rates (scale bars: 10 µm). (B) Seeded aligned PCL scaffolds with high growth rates form a monolayer over the whole surface (scale bars: 20 µm). FESEM, field emission scanning electron microscopy; PCL, polycaprolactone.

In terms of cell adhesion and proliferation, aligned nanofibrous scaffolds were better than random ones. Random PCL scaffold after 22 days showed a slower rate of cell growth; furthermore, the FESEM image also showed nonuniform proliferation when compared to the FESEM images of the aligned PCL scaffold seeded with cells (Figure 6).

## 4. Discussion

The journey of tissue regeneration and tissue or organ substitutes using advanced techniques or materials is planned to help in many diseases or artificial implantation, although this research may still require further investigation to restore tissues with all their components and structures, which may indicate the introduction of natural structures such as 20-hydroxyecdysone [24] and multivesicular vesicles as novel alternatives that still need improvement and research [25] or artificial structures such as PCL net to optimize the function of the substitutes. Multiple studies have hinted that periostin has a crucial role in regulating the development of periodontal tissue. Proteins like periostin, which are thought to be released by periodontal fibroblasts, are widely distributed throughout the ECM. After periodontal surgery, the PDL and alveolar bone may regenerate with the help of periostin, the future of periostin as an agent to stimulate periodontal tissue regeneration appears bright [26].

The biocompatibility of suggested biomaterials is essential before any real application or use; this can be studied in different ways. In order to conduct clinical risk assessments, ISO 14971 and ISO 10993-1 are used. As an illustration, the ISO 10993 series presents instructions for the selection of the appropriate methodologies to assess the toxicity of medical equipment, which includes dental materials. There are 20 separate components to this standard [27]. In vitro cytotoxicity assays are the initial stage in the evaluation of dental materials, just like they are with other substances. In addition to cell viability tests, cell interaction with these materials gives reliable results when compared with other laboratory tests. The surface properties of a biomaterial during application are just one of many variables that affect cells' favorable interactions with it. Scaffolds are primarily used in tissue engineering as a platform for cell adhesion and tissue regeneration and as a first supporting structure [28]. The ideal nanofiber scaffold should promote the development of new tissue and resemble the properties of PDL tissue. It is essential that the scaffold match, as closely as possible, the components of the target structure or tissue to be easily continued or integrated with the PDL tissue. Therefore, it is normal practice to enhance tissue restoration by selectively altering prefabricated scaffolds, considering the composition of the treated tissue. The architectural characteristics of scaffolds that are crucial for the effectiveness of the therapy must be thoroughly analyzed [2]. Using nanofiber scaffolds as opposed to microfiber scaffolds has led to comparable outcomes. The nanofibrous structures preserve cell shape while increasing ECM production. Nonetheless, it should be highlighted that pore size, in addition to fiber size and porosity, is a significant determinant [29]. The current study is investigating PCL scaffolds of nanofibers with different alignments that were made and comparing which is better to form PDL tissue: aligned fibers or randomly aligned fibers. Moreover, this study used plasma techniques to increase cell attachment on the scaffolds.

As shown in the microscopic images, the PCL nanofibers are highly porous, making them a good candidate for tissue engineering [30]. It was discovered that human cells could grow on aminolyzed PCL scaffolds and did so much more quickly than on plain PCL scaffolds. This was achieved by surface emodifying the PCL nanofibers using the wet chemistry method of aminolysis [31]. The surface's wettability can show how cells first interact with it and how they interact with it again later on. To treat PCL nanofibers, plasma was used, which increased their wettability and decreased the contact angle of groups, both randomly and in a straight line. This is why oxygen-containing functional groups were added after argon plasma treatment and nitrogen-containing functional groups were added after nitrogen plasma treatment. As claimed by Asadian et al. [32], this chemical change does not follow the arrangement of the nanofibers in the scaffold since this result was the same or similar whether the scaffold was in a well-aligned or random architecture. Similar findings were obtained from the surface characterization, which suggests that the plasma treatment contributed to the introduction of novel functional and polar groups on the fiber surface. This result disagrees with Sooriyaarachchi et al. [33], who assumed that there were differences in the contact angle between random or aligned fibers, although the variation in the measurement of contact angle in the groups could be attributed to the differences in porosity.

Results indicated that aligned nanofibers supported the growth of the PDLMCs more than the random type; this result disagrees with Jani, Al-Ameer, and Jawad [14], who showed significant improvement in cell proliferation opposite to the random nanofibers than the aligned one; this could be attributed to the type of mesenchymal cell used or duration of cell proliferation since Jani, Al-Ameer, and Jawad [14] measured cell proliferation up to 5 days only [8].

Plasma-treated PCL-aligned nanofiber scaffolds had cells that grew normally and had a spindle-shaped shape. Cells were oriented in different directions on the random nanofiber scaffold (Figure 6A), while for aligned scaffold nanofibers, the cells were arranged in the same direction and clustered around the aligned fibers in a longitudinal fashion (Figure 6B). This could be attributed to the orderliness of the nanofibers, which gave a good disposition for the cells to be attached and proliferate; on the other hand, the fashion of nanofiber distribution in a random manner creates pores with diffusion and size range, as well as roughness, which are not favorable for cell attachment and proliferation, although there were no significant differences considering wettability between aligned and random nanofiber scaffolds.

The average porosity of the fabricated nanofiber mats was 50%–60%, which probably increased the rate of attachment of the cells in comparison to the 3D printed scaffold without fibers. It can be attributed to the enhancement of the surface area and binding site. When the porosity increased, the attachment of the cells also increased when compared to a small porosity structure [34–37]. Nanosized structures mimic ECM structure efficiently, as well as enhancing the spreading of the cells and penetration. Thus, the fabrication of nanofibers can circumvent these constraints, resulting in bigger pore diameters, better cellular differentiation, and ECM production [38]. However, when the diameter of the random electrospun scaffold is increased, porosity is reduced. Moroni et al. [39] expressed this and reported that cell attachment and proliferation were reduced when the fiber diameter was increased. Aligned nanofibrous scaffolds have been proven to imitate the ECM and induce periodontal tissue regeneration. Low-porosity or high-density electrospun nanofiber scaffolds frequently result in limited cell permeability and restrict nutrition penetration to the deep zone of the tissue. In order to reinforce and expand the permeability and transport capacity of biomaterials through scaffold fibers, several alterations in fiber size and density have been employed. Injuries to the meniscal hoop structure in rabbits have been significantly repaired when parallel PCL strands and synovial-derived stem cells are used [2]. Furthermore, Shafiee et al. [40] demonstrated that aligned nanofibrous scaffolds outperformed randomly oriented scaffolds. The results of the study showed that aligned scaffolds led to bipolar extension along the fiber and a lot more chondrogenesis markers being expressed [40]. So, researchers have tried out different fiber sizes and densities to make biomaterials more permeable and increase their ability to move around. This is done by using scaffold fibers. Combining parallel PCL strands with stem cells was shown to help repair the meniscal hoop in rabbits in a big way [41]. Patient safety is the priority of any treatment modifications; therefore, benefit–risk analysis could help to serve patients effectively and successfully, as well as meet the optimal care for periodontal tissues and disease management [42]. Further in vivo studies may plan to coat the dental implant, attempting to mimic the vital PDL tissues [39, 43–45]. To reconstitute genuine PDL tissues and biomimetic implants, plasma-treated, random, and aligned PCL nanofibrous scaffolds were used to grow hPDLSCs. Only a small number of references are accessible in the literature; however, the new information presented here may help in tissue engineering using PDL cells.

## 5. Conclusions

In this study, it is concluded that plasma treatment is a successful method to increase the wettability of nanofibrous scaffolds, and this improvement in wettability is an influential factor affecting the hPDLSC's behavior. The aligned scaffold PCL increases cell proliferation compared to the random scaffold PCL. The aligned texture of the PCL nanofibrous scaffold is more appropriate as an artificial scaffold for hPDLSC regeneration in tissue engineering applications to guide PDL tissue regeneration than random PCL.

## Data Availability

Upon reasonable request, the corresponding author will provide access to the data supporting the conclusions of this study.
